# Monophasic pericardial synovial sarcoma in a turkish female patient: a very rare case with cyto-histopathological findings

**DOI:** 10.1186/s13019-023-02216-2

**Published:** 2023-05-11

**Authors:** Busra Yaprak Bayrak, Cigdem Vural, Huseyin Fatih Sezer, Aykut Eliçora, Yaprak Busra

**Affiliations:** 1 Department of Pathology, Faculty of Medicine, Kocaeli University, Kocaeli, Turkey; 2 Department of Thoracic Surgery, Faculty of Medicine, Kocaeli University, Kocaeli, Turkey

**Keywords:** Cytopathology, Histopathology, Pericardial synovial sarcoma, Soft tissue neoplasm

## Abstract

**Background:**

The aim was to present a 35-year-old female patient with diagnosis of monophasic primary pericardial synovial sarcoma (PSS) with cytopathological findings.

**Case Presentation:**

The case with back pain, palpitation and weakness, was diagnosed with pericardial effusion and suspicious mass adjacent to right heart in ultrasonography. Computerized tomography showed mass 12 × 11 × 6.5 cm in size, located in right mid-anterior pericardial area, with heterogeneous internal structure, heterogeneously contrasting right heart and prominent pressure on superior vena cava. Cytopathology of pericardial effusion showed monotonous cells with oval-spindle vesicular nuclei, less amphophilic cytoplasm, evenly distributed chromatin and inconspicuous nucleoli. The pericardial mass was resected incompletely, spindle cell mesenchymal tumor with hypercellular fascicular structure and with infiltrative margins, containing a small amount of loose myxoid stroma, occasionally necrotic areas was observed histopathologically. Immunohistochemical positive reaction was for vimentin, Bcl-2, TLE-1. Accordingly, the case was diagnosed with monophasic PSS.

**Conclusions:**

This case of monophasic primary PSS was an extremely rare malignancy diagnosed with the cytopathological findings.

## Background

Synovial sarcoma accounts for 5–10% of all soft tissue sarcomas. The lesion is often observed as a soft tissue tumor in the extremities of young adults. Localization of synovial sarcoma in the pericardium has rarely been reported in the literature [1]. There have been less than 20 cases of primary PSS published in the last decade [2–4]. Among the cases of synovial sarcoma, monophasic PSS is an extremely rare malignant tumor that is challenging to diagnose and unpredictable. Early diagnosis and subsequent aggressive multimodal treatment are crucial to successfully manage a patient with this condition [4]. The purpose of this report is to present the diagnostic and cytopathological experiences of a young patient with monophasic PSS.

## Case presentation

**Fig. 1:**
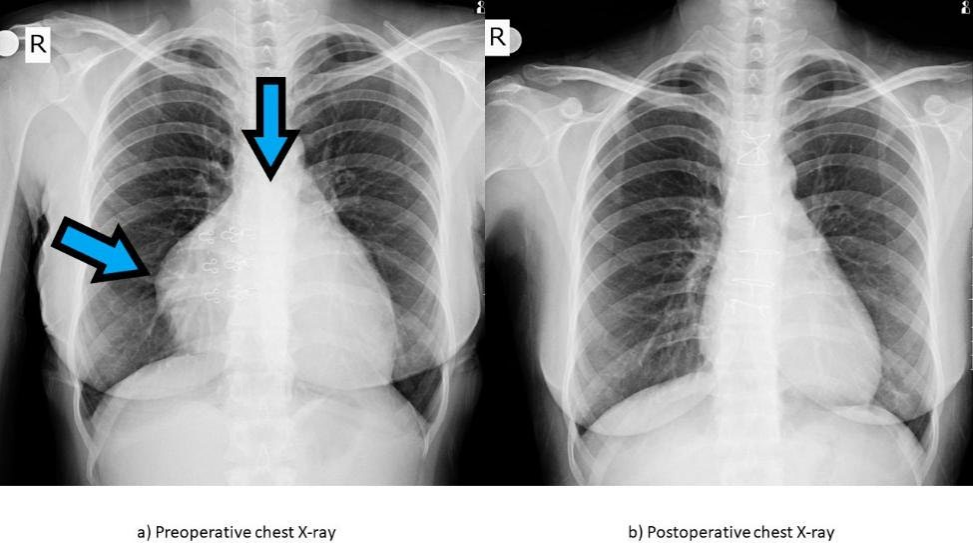
(a) Preoperative chest X-ray showing the mass in the right pericardiac and mediastinal area. (b) Postoperative chest X-ray showing the shrinking mass

**Fig. 2:**
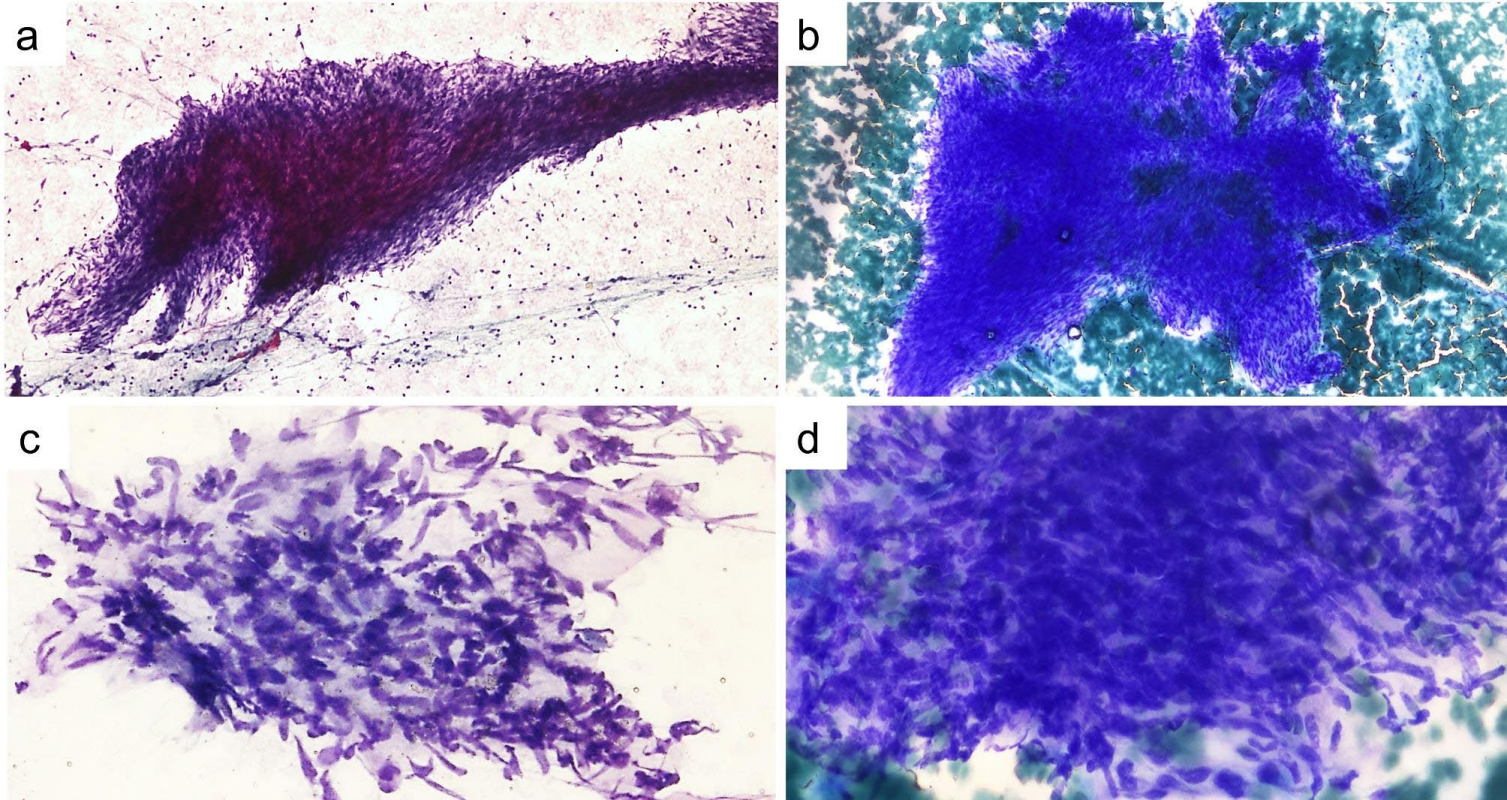
(a) Preoperative thoracic tomography of the heterogeneous mass lesion in the pericardial area, located in the right mid-anterior side, compressing the heart but not invaded into the superior vena cava or another vascular and cardiac region. (b) Postoperative thoracic tomography at 2nd year of operation (c) Postoperative thoracic tomography at 3rd year of operation showing the progress in mass size and invasion into adjacent regions

A 35-year-old female patient arrived at our department complaining of back pain, heart palpitations, and fatigue due to the large size of pericardial mass causing a pressure symptom. No symptoms of hypertension, fever, extrasystoles were found on physical examination, and no arrhythmia or rhythm disturbances were found on the electrocardiography. No previous medical and family history was declared by the patient. A chest X-ray showed that the patient’s cardiothoracic ratio was enlarged and that her cardiophrenic sinus was obscured on the right side (see Fig. 1). A transthoracic echocardiography indicated pericardial effusion, tricuspid, and pulmonary insufficiencies and showed a large echogenic mass involving the pericardium beside the right heart. Pericardiocentesis was performed, and a cytologic examination of the bloody pericardial fluid obtained though fine needle aspiration (FNA) showed monotonous cells with oval-spindle vesicular nuclei with less amphophilic cytoplasm, evenly distributed chromatin, and inconspicuous nucleoli that formed groups in hypocellular samples (see Fig. 2). Overlapping nuclei were observed locally. During pre-diagnosis, a spindle cell mesenchymal tumor with uncertain malignant potential was considered. Adequate sample cannot be obtained from aspiration for cell block preparation.

**Fig. 3:**
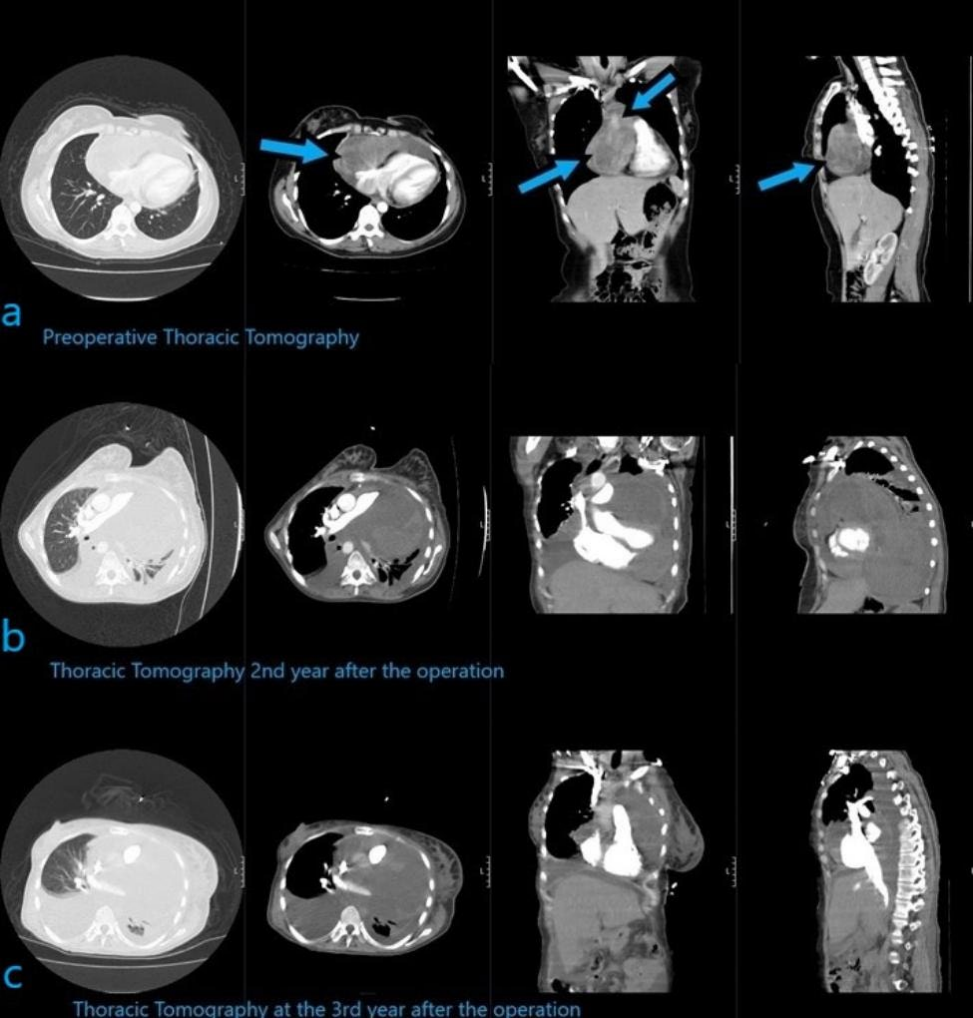
Light microscopic images of synovial sarcoma showing (a) spindle like cells clustered in the background with abundant stripped nuclei, scattered mast cells and abundant foamy macrophages (PAP, x 100); (b) spindle tumor cells form clusters with storiform arrangement (MGG, x100); (c) monotonous cells with scant amphophilic cytoplasm, ovoid to spindled vesicular nuclei with evenly dispersed chromatin and inconspicuous nucleoli (PAP, x400); (d) Nuclei close enough to overlap with adjacent nuclei (MGG, x400)

A contrasted CT scan of the chest showed a heterogeneously enhanced lesion measuring 12 cm x 11 cm x 6.5 cm located in the right mid-anterior of the pericardial area (see Fig. 3). The mass had a heterogeneous internal structure, creating significant pressure on the right heart and superior vena cava. There was no radiological space between the mass and the right heart, and no significant vascular, cardiac, or thoracic wall invasions were observed. No lymph node involvement was observed in the mediastinal or hilar regions. The trachea, including both main bronchi and bronchial trees, was open. Neither an active infiltrative appearance nor any nodular lesions could not be seen in the lung parenchyma (see Fig. 3). All radiological findings were indicative of either a pericardial fibroma or a pericardial sarcoma, both of which were noted in the differential diagnosis. On positron emission tomography (PET)-CT imaging, only the pericardial mass showed increased F-18 fluorodeoxyglucose (FDG) uptake with a maximum standard uptake of 5.3. No lymph node metastasis was detected radiologically.

After the radiological examinations, the pericardial mass was decided to be resected. During thoracotomy, the pericardial mass was found to invade the ventricular wall and main vascular structures, therefore, the mass was resected incompletely. Due to the location, extent and nature of the tumor, debulking surgery was performed and R0 surgery could not be performed. Macroscopically, any palpable lymph node was not detected during the surgery.

**Fig. 4:**
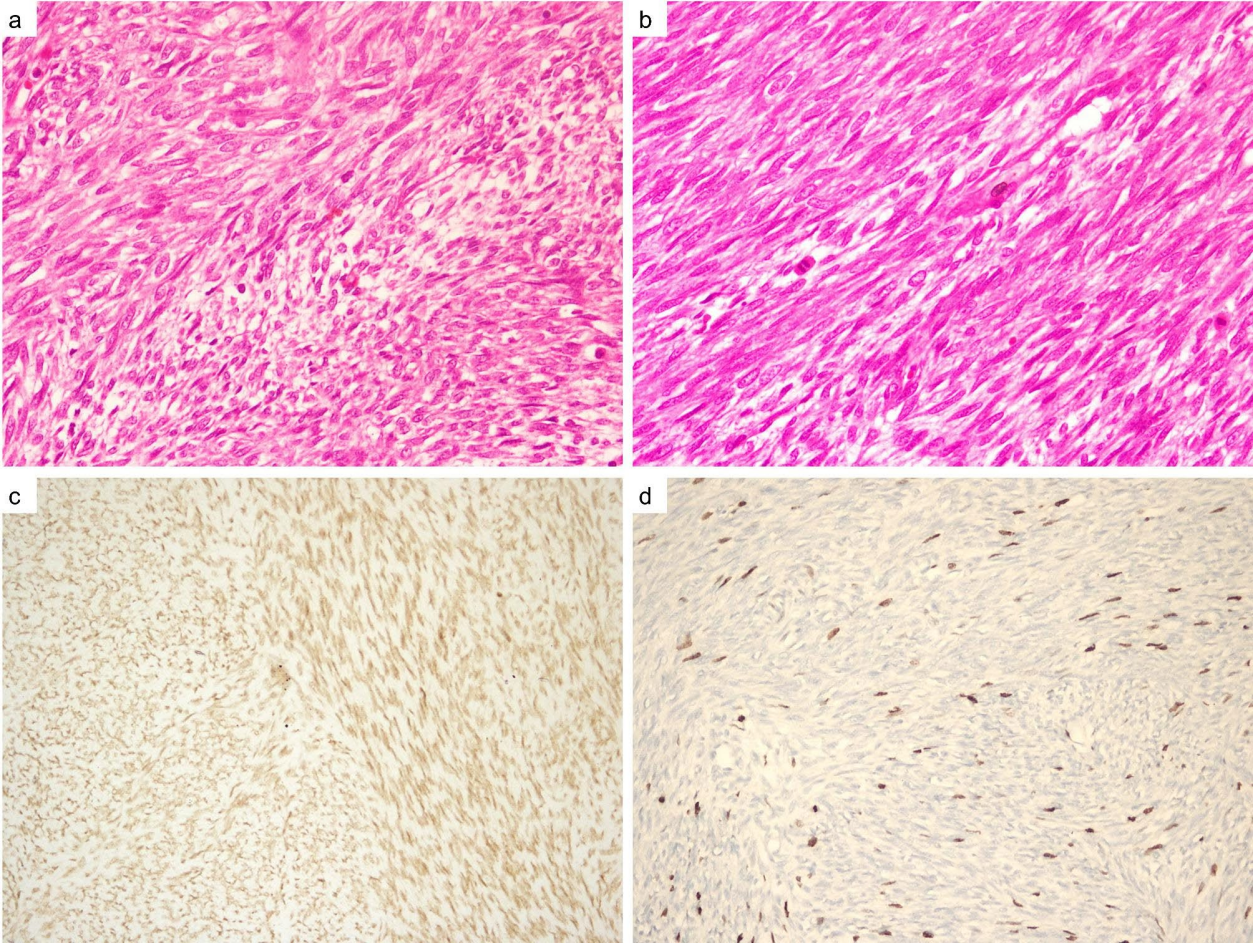
(a) Monotonous cells with oval to spindle shaped nuclei showing storiform pattern and admixed with various inflammatory cells (H & E, ×200). (b) Neoplastic cells with fine granular nuclei, inconspicuous nucleoli and scant cytoplasm, some of the cells including the mitotic figures (H and E, ×200). (c) Immunohistochemical expression of transduction-like enhancing protein 1 (TLE1) in tumor cells (×100). (d) Immunohistochemical expression of Ki-67 in tumor cells (×100)

Histopathologically, a spindle cell mesenchymal tumor with infiltrative limited hypercellular fascicular structure containing a small amount of loose myxoid stroma hyalinized, and necrotic areas showing mild to moderate pleomorphism were observed in fragmented mass samples (see Fig. 4a). A nuclear palisade was noted in the neoplastic cells, and the mitotic rate was 18 mitotic cells per 10 high power fields (see Fig. 4b). Mesothelioma, fibrosarcoma, a solitary fibrous tumor, leiomyosarcoma, and synovial sarcoma were all included as possibilities in the differential diagnosis. Immunohistochemical staining showed a positive reaction with vimentin, Bcl-2, TLE-1 (see Fig. 4c), and a negative reaction with pan-cytokeratin, HBME-1, calretinin, CD99, actin, desmin, CD34, STAT6, and S100. The Ki67 proliferation index was increased in the tumor (see Fig. 4d). A sample was sent to an external center where molecular studies could be performed for genetic analysis, and a real-time polymerase chain reaction (RT-PCR) test detected SYT-SSX1 fusion. With these findings, the patient was given a definitive diagnosed of monophasic synovial sarcoma.

Postoperative radiotherapy and chemotherapy were advised but refused by the patient at first place. On postoperative 4th month, the patient decided to undergo the oncological therapies, however, a six-week pregnancy was detected, therefore, only radiotherapy was applied due to the enlargement of pericardial mass. The chemotherapy was postponed for one year. After the term delivery, the patient suffered from the respiratory distress due to enlargement of the mass in the pericardial region to 14 cm in diameter, therefore, six cycles of doxorubicin and ifosfamide combination were administered. An emboli in the left main pulmonary artery, a pleural effusion in the right hemithorax, and a metastatic mass in the liver were detected. The patient whose respiratory parameters deteriorated was followed up in the intensive care unit. During this follow-up, the patient, who had myocardial infarction 21 months after the operation, died.

## Discussion

Primary pericardial sarcomas are a group of extremely rare malignancies that constitute approximately 10–15% of all primary cardiac sarcomas. These aggressive sarcomas are classified into histopathological subtypes, including fibrosarcoma, angiosarcoma, undifferentiated sarcoma, leiomyosarcoma and, synovial sarcoma [5]. Primary PSS has a poor prognosis compared to other types of sarcomas. Due to its rarity, the literature on the cytology of primary PSS is insufficient, making differential diagnoses difficult. It appears that this case of monophasic primary PSS which was an extremely rare malignancy diagnosed with the cytopathological findings. The cytology of a very limited number of cases with PSS has been studied in the literature, but none of them have been able to obtain sufficient numbers of cells by aspiration. Therefore, FNA cytology results could not be presented [6, 7].

Examinations involving PSS include a chest X-ray that may reveal the presence of a pericardiac mass in a widened mediastinum [8]. Echocardiographic findings include pericardial effusion or a thickened pericardium [9]. A CT scan determines the location of a tumor with either a local invasion or distant metastasis [10]. Chapra et al. (2021) presented the radiological findings of a large, heterogeneously enhancing pericardial mass with pericardial effusion in a patient diagnosed with primary PSS [2]. Recently, Luo et al. (2022) presented a young patient with a mediastinal mass noted in initial CT images, but the pathological examination and related auxiliary examinations of pericardial effusion did not show any tumor cells or related markers in the pleural fluid [11]. In our case, the clinical and radiological findings of the patient were supported by the histopathological and cytopathological findings. The patient was followed-up for three years, but the tumor had grown, aggravating the patient’s condition. She has recently been placed in intensive care.

Malignant pericardial effusions represent an advanced stage of malignancy and are less common than pleural or peritoneal effusions. Therefore, the clinical and cytopathologic features of these cases have rarely been reported. Studies conducted on pericardial effusions are relatively rare and have been limited to case reports and small series. Occasionally, a cytology examination cannot help to differentiate reactive mesothelial cells from malignant mesothelial or metastatic cells [12]. If a cytogenetic examination is not available, a cell block from aspiration material can be obtained, and an immunohistochemical analysis of the block can be performed for a differential diagnosis of PSS. In our case, no cell block was obtained, and an immunohistochemical analysis of the pericardiocentesis material could not be performed. However, resection material was obtained a week later from a cytological examination, and an immunohistochemical analysis of this material was performed. According to the findings of this analysis, the patient was definitively diagnosed with monophasic primary PSS, and multimodality therapy consisting of chemoradiotherapy was managed according to this diagnosis.

The biphasic type of PSS that is composed of an epithelial component and a spindle-cell component is more common than the monophasic type composed of only spindle-cells and poorly differentiated small round cells clustered in a fascicular pattern [6, 13]. Consistent with the literature, the histopathological findings of our patient with a monophasic variant of PSS showed similar cells in some areas with myxoid background and large areas of focal necrosis. The tumor was mitotically active.

The differential diagnosis of PSS includes various other mediastinal and pericardial tumors, including mesothelioma, fibrosarcoma, thymoma, metastatic carcinoma, and other soft tissue sarcoma. Tumor histology may show a similar appearance to PSS, so to differentiate PSS from other sarcomas, the immunohistochemistry for vimentin, BCL-2, CD99, and TLE1 are common positive markers [6]. The samples of our patient tested uniformly positive for vimentin, BCL-2, CD99, and TLE1 and tested negative for EMA, muscle markers (smooth muscle actin, desmin, and myogenin), the NSE neural marker, S-100, pankeratin (AE1/AE3), cytokeratin (CK5/6), CD57, p63 and FLI. This facilitated the correct differential diagnosis of PSS by excluding other possible soft tissue tumors. In some cases, tumor cells may also test positive for NSE and FLI1 [6]. Therefore, a cytogenetic examination of a sample is necessary for a definitive diagnosis.

## Conclusion

This case of PSS is an extremely rare malignancy, and it appears that it would be the first case with cytopathological findings to be published in the international literature. Because the progression of PSS is very rapid and because the morbidity and mortality rates of young people with PSS are relatively high, an aggressive surgical resection should be performed as soon as a patient is diagnosed. It should also be noted that FNAB is a helpful diagnostic method if it can be supported by molecular pathology.

## Data Availability

All data generated or analyzed during this study are included in this published article.
